# Microbial Phosphorus Solubilization and Its Potential for Use in Sustainable Agriculture

**DOI:** 10.3389/fmicb.2017.00971

**Published:** 2017-06-02

**Authors:** Elizabeth T. Alori, Bernard R. Glick, Olubukola O. Babalola

**Affiliations:** ^1^Department of Biological Sciences, Faculty of Agriculture, Science and Technology, North-West UniversityMmabatho, South Africa; ^2^Department of Biology, University of Waterloo, WaterlooON, Canada; ^3^Food Security and Safety Niche Area, North-West UniversityMmabatho, South Africa

**Keywords:** mineralization, phosphorus, soil nutrient management, soil microbes, solubilization

## Abstract

The use of excess conventional Phosphorus (P) fertilizers to improve agricultural productivity, in order to meet constantly increasing global food demand, potentially causes surface and ground water pollution, waterway eutrophication, soil fertility depletion, and accumulation of toxic elements such as high concentration of selenium (Se), arsenic (As) in the soil. Quite a number of soil microorganisms are capable of solubilizing/mineralizing insoluble soil phosphate to release soluble P and making it available to plants. These microorganisms improve the growth and yield of a wide variety of crops. Thus, inoculating seeds/crops/soil with Phosphate Solubilizing Microorganisms (PSM) is a promising strategy to improve world food production without causing any environmental hazard. Despite their great significance in soil fertility improvement, phosphorus-solubilizing microorganisms have yet to replace conventional chemical fertilizers in commercial agriculture. A better understanding of recent developments in PSM functional diversity, colonizing ability, mode of actions and judicious application should facilitate their use as reliable components of sustainable agricultural systems. In this review, we discussed various soil microorganisms that have the ability to solubilize phosphorus and hence have the potential to be used as bio fertilizers. The mechanisms of inorganic phosphate solubilization by PSM and the mechanisms of organic phosphorus mineralization are highlighted together with some factors that determine the success of this technology. Finally we provide some indications that the use of PSM will promote sustainable agriculture and conclude that this technology is ready for commercial exploitation in various regions worldwide.

## Introduction

Phosphorus (P) is one of the essential elements that are necessary for plant development and growth; it makes up about 0.2% of a plant’s dry weight. It is second only to nitrogen among mineral nutrients most commonly limiting the growth of crops (Azziz et al., 2012; Tak et al., 2012). On average, the phosphorus content of soil is about 0.05% (w/w); however, only 0.1% of this phosphorus is available for plant use (Zhu et al., 2011). Traditionally, the challenge of soil phosphorus deficiency is addressed by the application of phosphorus fertilizers. However, the majority of the applied fertilizer phosphorus is not available to plants and the addition of inorganic fertilizers in excess of the amount that is commonly employed to overcome this effect can lead to environmental problems such as, groundwater contamination and waterway eutrophication (Kang et al., 2011). It is therefore of great interest to investigate management strategies that are capable of improving phosphorus fertilization efficiency, increase crop yields and reduce environmental pollution caused by phosphorus loss from the soil.

Soil microorganisms enhance plant nutrient acquisition. They are involved in a wide range of biological processes including the transformation of insoluble soil nutrients (Babalola and Glick, 2012a). Some are capable of solubilizing and mineralizing insoluble soil phosphorus for the growth of plants. Apart from chemical fertilization, microbial P-solubilization and mineralization is the only possible way to increase plant-available phosphorus. In the natural environment numerous microorganisms in the soil and rhizosphere are effective at releasing phosphorus from total soil phosphorus through solubilization and mineralization (Bhattacharyya and Jha, 2012). This group of microorganisms are referred to as Phosphorus Solubilizing Microorganisms (PSM). Many species of soil fungi and bacteria are able to solubilize phosphorus *in vitro* and some of them can mobilize phosphorus in plants (Zhu et al., 2011). PSM increases the bioavailability of soil insoluble phosphorus for plant use (Zhu et al., 2011). They solubilize insoluble inorganic (mineral) phosphorus and mineralize insoluble organic phosphorus (Sharma et al., 2013). The salt-tolerant or halophilic soil microorganisms that also exhibit the ability to solubilize insoluble phosphorus facilitate the development of saline-alkali soil-based agriculture (Zhu et al., 2011).

The inoculation of soil or crop with phosphate solubilizing/mineralizing microorganisms is therefore a promising strategy for the improvement of plant absorption of phosphorus and thereby reducing the use of chemical fertilizers that have a negative impact on the environment (Alori et al., 2012).

## Phosphorus Solubilizing Microorganisms (PSM)

A large number of microbial organisms including bacteria, fungi, actinomycetes, and algae exhibit P solubilization and mineralization ability. Soil bacteria that have been reported to mobilize poorly available phosphorus via solubilization and mineralization include *Pseudomonas spp., Agrobacterium spp., and Bacillus circulans* (Babalola and Glick, 2012b). Other phosphorus solubilizing and mineralizing bacteria include various strains of *Azotobacter* (Kumar et al., 2014), *Bacillus* (Jahan et al., 2013; David et al., 2014), *Burkholderia* (Mamta et al., 2010; Zhao et al., 2014; Istina et al., 2015), *Enterobacter, Erwinia* (Chakraborty et al., 2009), *Kushneria* (Zhu et al., 2011), *Paenibacillus* (Fernández Bidondo et al., 2011), *Ralstonia, Rhizobium* (Tajini et al., 2012), *Rhodococcus, Serratia, Bradyrhizobium, Salmonella, Sinomonas*, and *Thiobacillus* (Postma et al., 2010; David et al., 2014).

The microbial fungi that function similarly include strains of *Achrothcium, Alternaria, Arthrobotrys, Aspergillus, Cephalosporium, Cladosporium, Curvularia, Cunninghamella, Chaetomium, Fusarium, Glomus, Helminthosporium, Micromonospora, Mortierella, Myrothecium, Oidiodendron, Paecilomyces, Penicillium, Phoma, Pichia fermentans, Populospora, Pythium, Rhizoctonia, Rhizopus, Saccharomyces, Schizosaccharomyces, Schwanniomyces, Sclerotium, Torula, Trichoderma*, and *Yarrowia* (Srinivasan et al., 2012; Sharma et al., 2013).

Soil fungi have been reported to be able to traverse long distances within the soil more easily than bacteria and may be more important to the solubilization of inorganic phosphate in soils as they typically produce and secrete more acids, such as gluconic, citric, lactic, 2-ketogluconic, oxalic, tartaric and acetic acid, than bacteria (Sharma et al., 2013). In addition, approximately 20% of actinomycetes could solubilize P, including those in the genera *Actinomyces, Micromonospora*, and *Streptomyces*. Algae such as cyanobacteria have also been reported to show P solubilization activity (Sharma et al., 2013).

## Benefits of Phosphorus Solubilizing Microorganism

**FIGURE 1:**
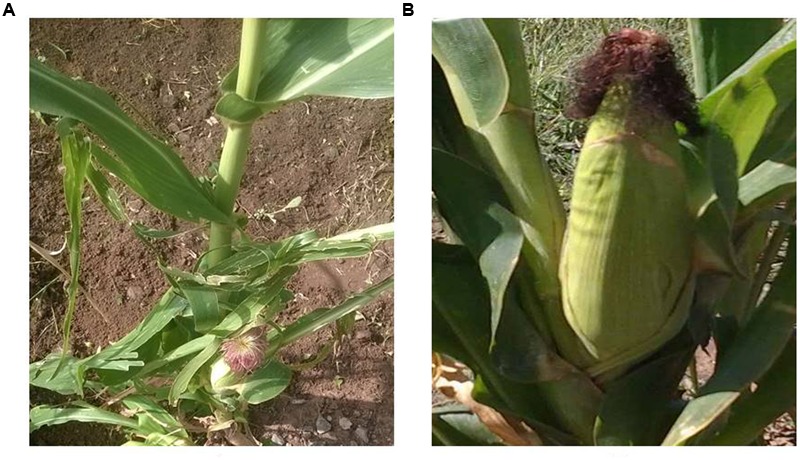
Biofertilizer effect of *Pseudomonas* sp. on maize. **(A)** Maize not inoculated with a PSM. **(B)** Maize inoculated with a PSM *Pseudomonas* sp.

For better utilization of the phosphorus accumulated in soils, PSMs that are capable of transforming insoluble phosphorus to soluble forms can function as biofertilizers. This increases the soluble phosphorus content (Zhu et al., 2012). The use of phosphorus biofertilizers is a promising approach to improving food production through enhancing agricultural yield as it is better to use an environmentally friendly approach (that is, a paradigm that emphasizes the use of biological soil amendments in place of chemicals) to solve the problems of infertile soil (Babalola and Glick, 2012a). **Figure 1** shows the effect of inoculation with a PSM (*Pseudomonas* sp.) on a maize plant. The growth of maize that was inoculated with PSM was improved compared to the control that was not inoculated. PSM act as biofertilizers by making otherwise unavailable P available to growing plants. Phosphorus solubilizing bacteria may also aid the growth of plants by stimulating the efficiency of biological nitrogen fixation, synthesizing phytohormones and enhancing the availability of some trace elements such as zinc and iron (Wani et al., 2007).

Many PSM inoculation studies have shown both improved plant yield and increased phosphorus uptake both in pot experiments and under field conditions. In a pot experiment where *Aspergillus niger* was used as a biofertilizer (using wheat husks with 20% perlite as carrier material) the soil colonization rate was 5.6 × 10^6^ spores g^-1^ soil (Wang et al., 2015). The benefits of adopting microbial management of the rhizosphere for sustainable agriculture production includes enhancing the bioavailability of phosphate to crops, stimulated roots and shoots growth, improved root and shoot length, and increased fresh and dry shoot weights, P-labeled phosphate uptake, and significant improvement of grain and dry matter yields (Rodríguez and Fraga, 1999). **Table 1** shows the effect of some PSM on a variety of crops.

**Table 1:** Effects of some PSM on crops.

PSM	Test crop	Result	Source
*A*spergillus *niger*	Wheat	Improved growth	Xiao et al., 2013
*Serratia* sp.	Wheat	Increased growth	Swarnalakshmi et al., 2013
*Aspergillus awamori*S29	Mung bean	Increased plant growth, total P content, and plant biomass	Jain et al., 2012
*Burkholderia gladioli*	Sweetleaf	Increased plant growth	Mamta et al., 2010
Pseudomonas aeruginosa	Chinese cabbage	Increased total weight and total length	Wang et al., 2010
*P. putid*a	Moss	Increased growth	Tani et al., 2011
*Azotobacter chroococcum, Saccharomyces cerevisiae*, and *Bacillus megaterium*	*Moringaoleifera*	Increased shoot and root lengths, increased shoot and root dry weights, increased vitamin C and protein content g/g dry weight leaves	Zayed, 2012
*Burkholderia gladioli*	Oil palm	Increased growth and phosphate uptake	Istina et al., 2015
*Aspergillus niger Penicillium aculeatum*	Chinese cabbage	Increased growth	Wang et al., 2015
*Bacillus* sp. and *Pseudomonas* sp.	Sesame	Increased seed yield	Jahan et al., 2013
*Bacillus thuringiensis*	Rice	Increased shoot length	David et al., 2014
*Pseudomonas striata* and *Glomus fasciculatum*	Soybean-wheat	Better root property and increased grain yield	Mahanta et al., 2014
*Burkholderia cepacia*	Maize	Improved plant growth	Zhao et al., 2014
*Azotobacter chroococcum* and *Bacillus subtilis*	Wheat	Enhanced productivity of wheat	Kumar et al., 2014
*P. favisporus* TG1R2	Soybeans	Increased dry biomass	Fernández Bidondo et al., 2011
*Rhizobium tropici* CIAT899	Beans	Enhanced increased; nodule number, nodule mass, shoot dry weight, and root growth	Tajini et al., 2012

Phosphate Solubilizing Microorganisms have considerable synergistic effect on the growth and development of crops (Tallapragada and Gudimi, 2011). Besides solubilizing P, some PSM also demonstrate potential as biocontrol agents against some plant pathogens. PSM manage the pathogens by producing antifungal compounds (such as PAL, phenolics and flavonoids), siderophores, antibiotics, hydrogen cyanide and lytic enzymes all of which enhance inhibition of the growth of plant pathogens.

Phosphate Solubilizing Microorganisms technology improves the fertility and agricultural use of saline-alkaline soil without causing any environmental or health hazard that accompanies the continuous use of synthetic fertilizers. *Kushneria* sp. YCWA18, a strain that is capable of solubilizing both inorganic phosphorus and organo-phosphorus has also demonstrated moderate halophilic properties and can be used in the development of saline-alkaline based agriculture (Zhu et al., 2011). *Aerococcus* sp. strain PSBCRG1-1, *Pseudomonas aeruginosa* strain PSBI3-1, *A. terreus* strain PSFCRG2-1 and *Aspergillus* sp. strain PSFNRH-2 were all shown to solubilize tricalcium phosphate at different NaCl concentrations (Srinivasan et al., 2012). The PSM *Burkholderia cepacia* promoted the growth of maize plants in the presence of NaCl concentrations of up to 5% (Zhao et al., 2014). These organisms all have potential as biofertilizers in saline-alkaline soil based agriculture. In one set of experiments, for bacterial solubilization, increases in NaCl concentration up to 0.8 M resulted in an increase in the percentage of phosphorus released but it declined thereafter. On the other hand, with increases in NaCl concentration the amount of P released among phosphate solubilizing fungi was found to decrease throughout the incubation periods (Srinivasan et al., 2012).

## Mechanisms of Inorganic Phosphate Solubilization by PSM

**FIGURE 2:**
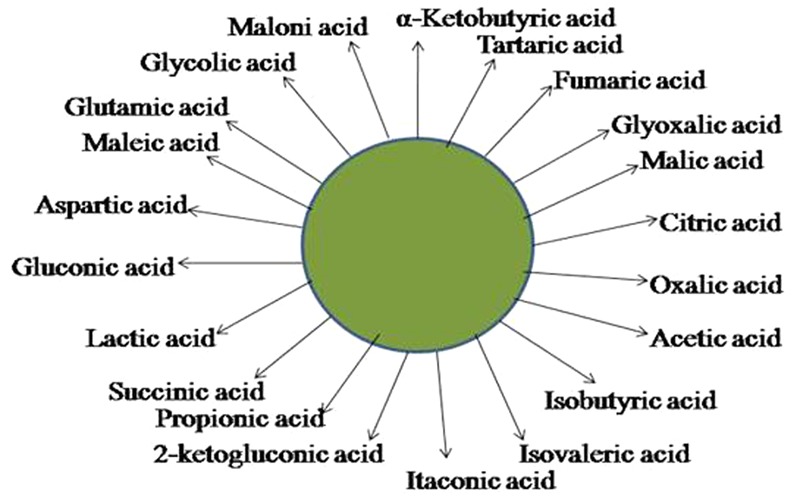
Schematic representation of the organic acids that may be produced by PSM and used to solubilize inorganic forms of phosphate.

A number of theories explain the mechanism of inorganic phosphate solubilization. As observed in many experiments, the principal mechanism is the production of mineral dissolving compounds such as organic acids, siderophores, protons, hydroxyl ions and CO2 (Rodríguez and Fraga, 1999; Sharma et al., 2013). Organic acids produced as described in **Figure 2** together with their carboxyl and hydroxyl ions chelate cations or reduce the pH to release P (Seshachala and Tallapragada, 2012); The organic acids are produced in the periplasmic space by the direct oxidation pathway (Zhao et al., 2014). The excretion of these organic acids is accompanied by a drop in pH that results in the acidification of the microbial cells and the surroundings, hence, P ions are released by substitution of H^+^ for Ca^2+^ (Goldstein, 1994). Surprisingly, Asea et al. (1988) discovered that no correlation exists between the pH and the amount of P solubilized. Hence Illmer and Schinner (1995) proposed the theory of acidification by H^+^. They explained that H^+^ released is associated with cation assimilation. For example, assimilation of NH_4_^+^ together with H^+^ excretion brings about P solubilisation (Illmer and Schinner, 1995). An alternative mechanism to organic acid production for solubilization of mineral phosphates is the release of H^+^ to the outer surface in exchange for cation uptake or with the help of H^+^ translocation ATPase (Rodríguez and Fraga, 1999). It was also reported that the assimilation of NH4^+^ within microbial cells is accompanied by the release of protons and this results in the solubilization of phosphorus without the production of any organic acids (Sharma et al., 2013). Of all the organic acids, gluconic acid is the most frequent agent of mineral phosphate solubilization; it chelates the cations bound to phosphate, thus making the phosphate available to plants. Gram-negative bacteria solubilize mineral phosphate by direct oxidation of glucose to gluconic acid (Goldstein, 2000). Pyrroloquinoline quinone (PQQ) acts as a redox cofactor in glucose dehydrogenases (GDH) resulting in phosphate solubilisation (Rodríguez et al., 2000).

Other mechanisms of mineral phosphate solubilization by microorganisms are the production of inorganic acids (such as sulphuric, nitric, and carbonic acids) and the production of chelating substances. It has, however, been reported that the effectiveness of the inorganic acids and the chelating substances in the release of phosphorus in soil is less than that of the organic acids. Kim et al. (1997b) therefore reiterate that organic acid production in P solubilization by PSM is not the sole reason for the increase in P concentration into culture medium. Furthermore, Mycorrhizal fungi effectively extend plant roots, aiding crop phosphorus nutrition by increasing the volume of soil from which phosphate may be absorbed (Browne et al., 2009).

Another mechanism of microbial phosphate solubilization reported in the literature is the liberation of enzymes or enzymolysis, the mechanism of P solubilization by PSM in a medium containing lecithin where the increase in acidity is caused by enzymes that act on lecithin and produce choline (Zhu et al., 2011).

## Mechanisms of Organic Phosphorus Mineralization

The major source of organic phosphorus in soil is the organic matter. The values of organic phosphorus in soil can be as high as 30–50% of the total P and soil organic P is largely in the form of inositol phosphate (soil phytate). Other organic P compounds that have been reported are: phosphomonoesters, phosphodiesters, phospholipids, nucleic acids, and phosphotriesters (Rodríguez and Fraga, 1999). In addition, large quantities of xenobiotic phosphonates (pesticides, detergent additives, antibiotics, and flame retardants) that are regularly released into the environment also contain organic P. Most of these organic compounds are high molecular-weight materials that are generally resistant to chemical hydrolysis and must therefore be bio-converted to either soluble ionic phosphate (Pi, HPO_4_^2-^, H_2_PO_4_^-^), or low molecular-weight organic phosphate, to be assimilated by the cell (Peix et al., 2001).

Phosphorus mineralization refers to the solubilization of organic phosphorus and the degradation of the remaining portion of the molecule. One important theory proposed by Halvorson et al. (1990) for the solubilisation of organic P is the sink theory. This refers to continuous removal of P that result in the dissolution of Ca-P compounds. Consequently, the decomposition of P in organic substrates is consistently correlated with the P content in the biomass of PSM (Dighton and Boddy, 1989). This biological process plays an important role in phosphorus cycling. Different groups of enzymes are involved in this. The first groups of enzymes are those that dephosphorylate the phosphor-ester or phosphoanhydride bond of organic compounds. They are non-specific acid phosphatases (NSAPs). The most studied among these NSAPs enzymes released by PSM, are the phosphomonoesterases also referred to as phosphatases (Nannipieri et al., 2011). These enzymes can either be acid or alkaline phosphomonoesterases (Jorquera et al., 2011). The pH of most soils where phosphate activities were reported ranges from acidic to neutral values. This signifies that acid phosphatases play the major role in this process (Rodríguez and Fraga, 1999).

Another enzyme produced by PSM in the process of organic P mineralization is phytase. This enzyme is responsible for the release of phosphorus from organic materials in soil (plant seeds and pollen) that are stored in the form of phytate. Phytate degradation by phytase releases phosphorus in a form that is available for plant use. Plants generally cannot acquire phosphorus directly from phytate, however, the presence of PSM within the rhizosphere may compensate for a plant’s inability to otherwise acquire phosphorus directly from phytate (Richardson and Simpson, 2011).

## Factors Influencing Microbial Phosphate Solubilization

The ability of PSM to transform insoluble organic and inorganic phosphorus is associated with, the nutritional richness of the soil, and the physiological and growth status of the organism. PSM from soils from environmental extremes such as saline-alkaline soils, soil with a high level of nutrient deficiency, or soil from extreme temperature environments have the tendency to solubilize more phosphate than PSM from soils from more moderate conditions (Zhu et al., 2011). There has been a conflicting report on the influence of temperature on phosphorus solubilization by microbes. White et al. (1997) found 20–25°C as the optimum temperature for maximum microbial phosphorus solubiliztion while 28°C was reported by Kang et al. (2002), and Varsha (2002). In addition, others including Kim et al. (1997a), Rosado et al. (1998), Johri et al. (1999), and Fasim et al. (2002), have recorded 30°C as the best temperature for P solubilization. Nahas (1996) and Nautiyal et al. (2000) reported P solubilization at extreme temperature of 45°C in desert soil while Johri et al. (1999) reported solubilization at a low temperature of 10°C.

Among other factors influencing microbial phosphate solubilization are interactions with other microorganisms in the soil, the extent of vegetation, ecological conditions, climatic zone soil types, plant types, agronomic practices, land use systems, and the soil’s physicochemical properties such as organic matter and soil pH (Seshachala and Tallapragada, 2012). Phosphorus is solubilized faster in warm humid climates and slower in cool dry climates. A well-aerated soil will more readily permit rapid phosphorus solubilisation compared to a saturated wet soil. The land use system is the use that the farmland has been previously committed to, such as cropping or livestock activities or even mixed use. Recently, Zhang et al. (2014) reported that adding small amounts of inorganic phosphorus to the rhizosphere could drive phytic acid mineralization by bacteria and thereby improve plant phosphorus nutrition. Lime and compost, used as a soil improver, also had positive effects on phosphate solubilizers. Phosphorus Solubilizing Bacteria population richness and diversity, according to Azziz et al. (2012), were more abundant and diverse following crop rotation. Soil rich in organic matter will favor microbial growth and therefore favors microbial phosphorus solubilisation. Soil pH values between 6 and 7.5 are best for P-availability, this is because at pH values below 5.5 and between 7.5 and 8.5 limits P from becoming fixed by aluminum, iron, or calcium, and hence, not being available for plant use. A negative correlation was observed between the amount of phosphate solubilized by *B. cepacia* SCAUK0330 and the pH drop that is associated with this process. The pH drop leads to an increase in phosphate solubilization. At pH 3.12, 452 μg⋅mL^-1^ of phosphorus was solubilized, and when 154 μg⋅mL^-1^ of P was solubilized the pH value was 4.95 (Zhao et al., 2014). Research has also shown that microbial phosphate solubilization largely depends on the kinds of metabolite produced and its rate of release (Zhu et al., 2011).

## Future Prospects

As additional insights are gained regarding PSM and the mechanisms that they use, there is every reason to believe that the use of PSM as biofertilizers will likely improve their use, as effective and important components in the establishment of sustainable soil management systems. The focus of consumers of agricultural produce is on the health, quality and nutritional value of those products. Thus, the employment of PSM as biofertilizers is an option that can increase food production without imposing any health hazard, and at the same time conserve the environment. It is essential that researchers continue to learn more about PSM and, immediately, translate this knowledge into a form that can readily be used by farmers.

## Conclusion

This review has shown that phosphate-solubilizing microorganisms have tremendous potential as Bio-fertilizers. Mobilizing soil inorganic phosphate and increasing its bioavailability for plant use by harnessing soil PSM promotes sustainable agriculture, improves the fertility of the soil, and hence increases crop productivity. The use of PSM as microbial inoculants is a new horizon for better plant productivity. PSM technology can contribute to low-input farming systems and a cleaner environment. However, there is need to develop PSB technologies specific to various regions and this should be communicated to farmers in a relatively short time.

## Author Contributions

All authors listed, have made substantial, direct and intellectual contribution to the work, and approved it for publication.

## Conflict of Interest Statement

The authors declare that the research was conducted in the absence of any commercial or financial relationships that could be construed as a potential conflict of interest.

## Abbreviations

PSM: phosphate solubilizing microorganisms
